# 9-(4-Hy­droxy­phen­yl)-3,3,6,6-tetra­methyl-4,5,6,9-tetra­hydro-3*H*-xanthene-1,8(2*H*,7*H*)-dione

**DOI:** 10.1107/S160053681102527X

**Published:** 2011-07-02

**Authors:** Hoong-Kun Fun, Wan-Sin Loh, K. Rajesh, V. Vijayakumar, S. Sarveswari

**Affiliations:** a X-ray Crystallography Unit, School of Physics, Universiti Sains Malaysia, 11800 USM, Penang, Malaysia; b Organic Chemistry Division, School of Advanced Sciences, VIT University, Vellore 632 014, India

## Abstract

In the title compound, C_23_H_26_O_4_, the two cyclo­hexene rings adopt envelope conformations whereas the pyran ring adopts a boat conformation. In the crystal, pairs of inter­molecular O—H⋯O hydrogen bonds link the mol­ecules into inversion dimers.

## Related literature

For background to xanthene derivatives and their microbial activity, see: Jonathan *et al.* (1988 ▶); Hatakeyama *et al.* (1988 ▶); Shchekotikhin & Nikolaeva (2006 ▶); Hilderbrand & Weissleder (2007 ▶); Pohlers & Scaiano (1997 ▶); Knight & Stephens (1989 ▶); Reddy *et al.* (2010 ▶); Rathore *et al.* (2009 ▶); Rajesh *et al.* (2010 ▶); Mookiah *et al.* (2009 ▶). For ring conformations, see: Cremer & Pople (1975 ▶). For bond-length data, see: Allen *et al.* (1987 ▶). For a related structure, see: Odabaşoğlu *et al.* (2008 ▶). For the stability of the temperature controller used in the data collection, see: Cosier & Glazer (1986 ▶).
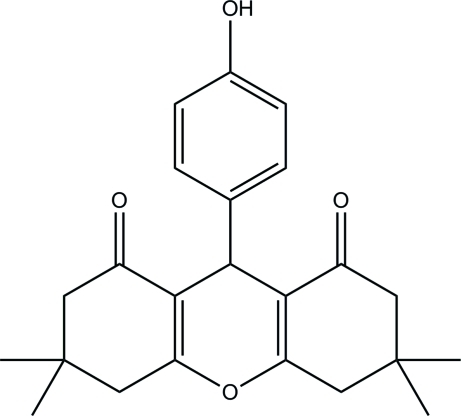


## Experimental

### 

#### Crystal data

C_23_H_26_O_4_
*M* *_r_* = 366.44
Triclinic, 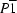

*a* = 9.3525 (1) Å
*b* = 10.2140 (1) Å
*c* = 11.6913 (1) Å
α = 67.271 (1)°
β = 76.119 (1)°
γ = 69.419 (1)°
*V* = 957.32 (2) Å^3^
*Z* = 2
Mo *K*α radiation
μ = 0.09 mm^−1^
*T* = 100 K
0.43 × 0.37 × 0.25 mm

#### Data collection

Bruker SMART APEXII CCD area-detector diffractometer
Absorption correction: multi-scan (*SADABS*; Bruker, 2009 ▶) *T*_min_ = 0.964, *T*_max_ = 0.979
31226 measured reflections
8415 independent reflections
7287 reflections with *I* > 2σ(*I*)
*R*_int_ = 0.019

#### Refinement

*R*[*F* ^2^ > 2σ(*F* ^2^)] = 0.039
*wR*(*F* ^2^) = 0.119
*S* = 1.05
8415 reflections
252 parameters
H atoms treated by a mixture of independent and constrained refinement
Δρ_max_ = 0.59 e Å^−3^
Δρ_min_ = −0.21 e Å^−3^

### 

Data collection: *APEX2* (Bruker, 2009 ▶); cell refinement: *SAINT* (Bruker, 2009 ▶); data reduction: *SAINT*; program(s) used to solve structure: *SHELXTL* (Sheldrick, 2008 ▶); program(s) used to refine structure: *SHELXTL*; molecular graphics: *SHELXTL*; software used to prepare material for publication: *SHELXTL* and *PLATON* (Spek, 2009 ▶).

## Supplementary Material

Crystal structure: contains datablock(s) global, I. DOI: 10.1107/S160053681102527X/is2739sup1.cif

Structure factors: contains datablock(s) I. DOI: 10.1107/S160053681102527X/is2739Isup2.hkl

Supplementary material file. DOI: 10.1107/S160053681102527X/is2739Isup3.cml

Additional supplementary materials:  crystallographic information; 3D view; checkCIF report

## Figures and Tables

**Table 1:** Hydrogen-bond geometry (Å, °)

*D*—H⋯*A*	*D*—H	H⋯*A*	*D*⋯*A*	*D*—H⋯*A*
O4—H1*O*4⋯O2^i^	0.851 (18)	1.910 (17)	2.7423 (9)	165.6 (17)

Symmetry code: (i) 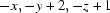.

**Fig. 1.**
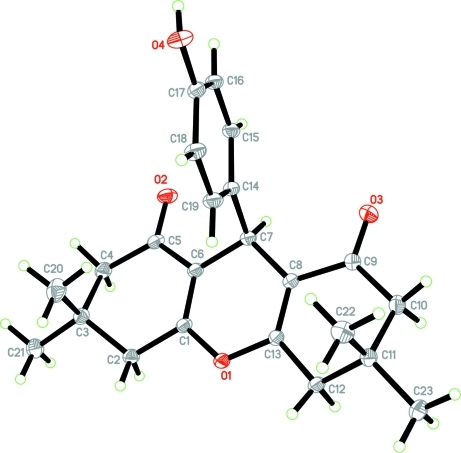
The molecular structure of the title compound, showing 50% probability displacement ellipsoids and the atom-numbering scheme.

**Fig. 2.**
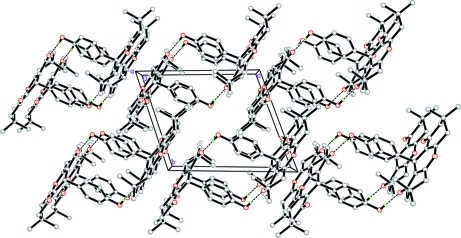
The crystal packing of the title compound, viewed along the a axis. H atoms not involved in the intermolecular interactions (dashed lines) have been omitted for clarity.

## supplementary crystallographic information

### Comment

Xanthenes are known for possessing various biological properties including antibacterial, antiviral and anti-inflammatory activities (Jonathan *et al.*, 1988). In particular, xanthenedione derivatives constitute a structural unit in several natural products (Hatakeyama *et al.*, 1988), and they are valuable synthons because of the inherent reactivity of the inbuilt pyran ring (Shchekotikhin *et al.*, 2006). Xanthene derivatives are also very useful and important organic compounds widely used as dye (Hilderbrand *et al.*, 2007), in laser technologies (Pohlers *et al.*, 1997), and fluorescent materials for visualization of biomolecules (Knight *et al.*, 1989). The structural resemblance of xanthenes to 1,4-dihydropyridines which was our area of interest (Reddy *et al.*, 2010; Rathore *et al.*, 2009; Rajesh *et al.*, 2010; Mookiah *et al.*, 2009), and which can function as calcium channel blockers made us to focus on xanthenes and its synthesis.

In the title compound (Fig. 1), the cyclohexene (C1–C6 & C8–C13) and the pyran (O1/C1/C6–C8/C13) rings are not planar, having puckering amplitudes, Q of 0.4840 (8), 0.4538 (8) and 0.1049 (8) Å, respectively. The cyclohexene rings (C1–C6 & C8–C13) adopt envelope conformations [φ = 118.78 (11)° and Θ = 58.70 (9)°; φ = 3.56 (12)° and Θ = 120.92 (10)°] whereas the pyran ring adopts a boat conformation [φ = 355.6 (5)° and 114.4 (4)°; Cremer & Pople, 1975). Bond lengths and angles are comparable to the related structure (Odabaşoğlu *et al.*, 2008).

In the crystal packing (Fig. 2), intermolecular O4—H1O4···O2 hydrogen bonds (Table 1) link the molecules into dimers.

### Experimental

A mixture of *p*-hydroxybenzaldehyde (0.5 g, 8 mmol) and 5,5-dimethyl-1,3-cyclohexanedione (1.15 g, 1.6 mmol) were mixed along with 15 ml of ethylene glycol and then heated at 70 °C for about 1.5 h. The progress of the reaction was monitored by TLC. After confirming that the reaction was completed, the reaction mixture was allowed to cool to room temperature and poured it onto water. The solid obtained was filtered, dried and recrystalized from chloroform/methanol (1:1) to yield colourless crystals (*m.p.* 519–521 K).

### Refinement

Atom H1O4 was located in a difference Fourier map and was refined freely [O—H = 0.851 (17) Å]. The remaining H atoms were positioned geometrically (C—H = 0.96 or 0.98 Å) and were refined using a riding model, with *U*_iso_(H) = 1.2 or 1.5*U*_eq_(C). A rotating group model was applied to the methyl groups.

### Crystal data

**Table:**

C_23_H_26_O_4_	*Z* = 2
*M**_r_* = 366.44	*F*(000) = 392
Triclinic, *P*1	*D*_x_ = 1.271 Mg m^−^^3^
Hall symbol: -P 1	Mo *K*α radiation, λ = 0.71073 Å
*a* = 9.3525 (1) Å	Cell parameters from 9935 reflections
*b* = 10.2140 (1) Å	θ = 2.3–35.1°
*c* = 11.6913 (1) Å	µ = 0.09 mm^−^^1^
α = 67.271 (1)°	*T* = 100 K
β = 76.119 (1)°	Block, colourless
γ = 69.419 (1)°	0.43 × 0.37 × 0.25 mm
*V* = 957.32 (2) Å^3^	

### Data collection

**Table:**

Bruker SMART APEXII CCD area-detector diffractometer	8415 independent reflections
Radiation source: fine-focus sealed tube	7287 reflections with *I* > 2σ(*I*)
graphite	*R*_int_ = 0.019
φ and ω scans	θ_max_ = 35.2°, θ_min_ = 1.9°
Absorption correction: multi-scan (*SADABS*; Bruker, 2009)	*h* = −15→15
*T*_min_ = 0.964, *T*_max_ = 0.979	*k* = −14→16
31226 measured reflections	*l* = −17→18

### Refinement

**Table:**

Refinement on *F*^2^	Primary atom site location: structure-invariant direct methods
Least-squares matrix: full	Secondary atom site location: difference Fourier map
*R*[*F*^2^ > 2σ(*F*^2^)] = 0.039	Hydrogen site location: inferred from neighbouring sites
*wR*(*F*^2^) = 0.119	H atoms treated by a mixture of independent and constrained refinement
*S* = 1.05	*w* = 1/[σ^2^(*F*_o_^2^) + (0.0652*P*)^2^ + 0.1943*P*] where *P* = (*F*_o_^2^ + 2*F*_c_^2^)/3
8415 reflections	(Δ/σ)_max_ = 0.001
252 parameters	Δρ_max_ = 0.59 e Å^−^^3^
0 restraints	Δρ_min_ = −0.21 e Å^−^^3^

### Special details

**Table:**

Experimental. The crystal was placed in the cold stream of an Oxford Cryosystems Cobra open-flow nitrogen cryostat (Cosier & Glazer, 1986) operating at 100.0 (1) K.
Geometry. All e.s.d.'s (except the e.s.d. in the dihedral angle between two l.s. planes) are estimated using the full covariance matrix. The cell e.s.d.'s are taken into account individually in the estimation of e.s.d.'s in distances, angles and torsion angles; correlations between e.s.d.'s in cell parameters are only used when they are defined by crystal symmetry. An approximate (isotropic) treatment of cell e.s.d.'s is used for estimating e.s.d.'s involving l.s. planes.
Refinement. Refinement of *F*^2^ against ALL reflections. The weighted *R*-factor *wR* and goodness of fit *S* are based on *F*^2^, conventional *R*-factors *R* are based on *F*, with *F* set to zero for negative *F*^2^. The threshold expression of *F*^2^ > σ(*F*^2^) is used only for calculating *R*-factors(gt) *etc*. and is not relevant to the choice of reflections for refinement. *R*-factors based on *F*^2^ are statistically about twice as large as those based on *F*, and *R*- factors based on ALL data will be even larger.

### Fractional atomic coordinates and isotropic or equivalent isotropic displacement parameters (Å^2^)

**Table:**

	*x*	*y*	*z*	*U*_iso_*/*U*_eq_	
O1	0.34521 (6)	0.83032 (6)	0.99915 (5)	0.01579 (9)	
O2	0.02841 (7)	1.17532 (6)	0.69020 (5)	0.02010 (11)	
O3	−0.10300 (7)	0.70038 (7)	1.02725 (5)	0.02242 (11)	
O4	0.16175 (7)	0.64603 (6)	0.49102 (5)	0.02133 (11)	
C1	0.30785 (8)	0.95394 (7)	0.89677 (6)	0.01442 (11)	
C2	0.42026 (8)	1.04139 (8)	0.85853 (6)	0.01674 (12)	
H2A	0.5217	0.9742	0.8762	0.020*	
H2B	0.3915	1.1068	0.9074	0.020*	
C3	0.42656 (8)	1.13402 (7)	0.71914 (6)	0.01599 (11)	
C4	0.26065 (9)	1.22259 (8)	0.69255 (7)	0.01843 (12)	
H4A	0.2241	1.2977	0.7326	0.022*	
H4B	0.2604	1.2733	0.6032	0.022*	
C5	0.14947 (8)	1.13060 (7)	0.73661 (6)	0.01591 (11)	
C6	0.18373 (8)	0.98961 (7)	0.83917 (6)	0.01459 (11)	
C7	0.08188 (8)	0.88931 (7)	0.87495 (6)	0.01454 (11)	
H7A	−0.0257	0.9474	0.8870	0.017*	
C8	0.12303 (8)	0.76682 (7)	0.99633 (6)	0.01441 (11)	
C9	0.02202 (8)	0.67001 (8)	1.06217 (6)	0.01635 (11)	
C10	0.07186 (8)	0.53779 (8)	1.17715 (7)	0.01825 (12)	
H10A	0.0404	0.4566	1.1776	0.022*	
H10B	0.0174	0.5639	1.2507	0.022*	
C11	0.24508 (8)	0.48275 (7)	1.18770 (6)	0.01565 (11)	
C12	0.30063 (8)	0.61720 (7)	1.16306 (6)	0.01557 (11)	
H12A	0.2626	0.6521	1.2341	0.019*	
H12B	0.4122	0.5866	1.1551	0.019*	
C13	0.24825 (8)	0.74137 (7)	1.04798 (6)	0.01417 (11)	
C14	0.10119 (8)	0.82609 (7)	0.77113 (6)	0.01428 (11)	
C15	−0.01107 (8)	0.87860 (7)	0.69164 (6)	0.01535 (11)	
H15A	−0.1004	0.9526	0.7027	0.018*	
C16	0.00783 (8)	0.82251 (7)	0.59602 (6)	0.01557 (11)	
H16A	−0.0677	0.8601	0.5431	0.019*	
C17	0.14000 (8)	0.70988 (7)	0.57955 (6)	0.01580 (11)	
C18	0.25476 (8)	0.65753 (8)	0.65697 (7)	0.01858 (12)	
H18A	0.3442	0.5839	0.6457	0.022*	
C19	0.23478 (8)	0.71605 (8)	0.75134 (6)	0.01734 (12)	
H19A	0.3120	0.6811	0.8023	0.021*	
C20	0.50082 (11)	1.03502 (10)	0.63814 (8)	0.02510 (15)	
H20A	0.5058	1.0956	0.5516	0.038*	
H20B	0.4405	0.9697	0.6530	0.038*	
H20C	0.6029	0.9775	0.6588	0.038*	
C21	0.52047 (10)	1.24087 (8)	0.69189 (7)	0.02181 (14)	
H21A	0.5222	1.3013	0.6053	0.033*	
H21B	0.6238	1.1854	0.7104	0.033*	
H21C	0.4745	1.3031	0.7429	0.033*	
C22	0.33171 (9)	0.40175 (8)	1.09396 (7)	0.02121 (13)	
H22A	0.2959	0.3178	1.1116	0.032*	
H22B	0.4399	0.3687	1.1007	0.032*	
H22C	0.3136	0.4680	1.0108	0.032*	
C23	0.27504 (9)	0.37755 (8)	1.32038 (7)	0.02137 (13)	
H23A	0.2417	0.2922	1.3380	0.032*	
H23B	0.2191	0.4278	1.3792	0.032*	
H23C	0.3831	0.3467	1.3271	0.032*	
H1O4	0.0906 (19)	0.6959 (18)	0.4441 (15)	0.049 (4)*	

### Atomic displacement parameters (Å^2^)

**Table:**

	*U*^11^	*U*^22^	*U*^33^	*U*^12^	*U*^13^	*U*^23^
O1	0.0185 (2)	0.0151 (2)	0.0139 (2)	−0.00786 (17)	−0.00582 (16)	0.00009 (16)
O2	0.0238 (3)	0.0185 (2)	0.0186 (2)	−0.00527 (19)	−0.01005 (19)	−0.00298 (18)
O3	0.0192 (2)	0.0266 (3)	0.0221 (2)	−0.0106 (2)	−0.00590 (19)	−0.0033 (2)
O4	0.0263 (3)	0.0223 (2)	0.0170 (2)	−0.0028 (2)	−0.00794 (19)	−0.00877 (19)
C1	0.0185 (3)	0.0136 (2)	0.0116 (2)	−0.0058 (2)	−0.0037 (2)	−0.0025 (2)
C2	0.0210 (3)	0.0167 (3)	0.0145 (3)	−0.0092 (2)	−0.0052 (2)	−0.0022 (2)
C3	0.0206 (3)	0.0154 (3)	0.0131 (2)	−0.0077 (2)	−0.0017 (2)	−0.0040 (2)
C4	0.0225 (3)	0.0157 (3)	0.0155 (3)	−0.0072 (2)	−0.0050 (2)	−0.0005 (2)
C5	0.0208 (3)	0.0147 (2)	0.0126 (2)	−0.0050 (2)	−0.0049 (2)	−0.0034 (2)
C6	0.0178 (3)	0.0143 (2)	0.0121 (2)	−0.0057 (2)	−0.0042 (2)	−0.0026 (2)
C7	0.0156 (3)	0.0156 (2)	0.0129 (2)	−0.0051 (2)	−0.00397 (19)	−0.0033 (2)
C8	0.0163 (3)	0.0155 (2)	0.0119 (2)	−0.0062 (2)	−0.00282 (19)	−0.0031 (2)
C9	0.0173 (3)	0.0177 (3)	0.0148 (3)	−0.0072 (2)	−0.0019 (2)	−0.0044 (2)
C10	0.0176 (3)	0.0173 (3)	0.0177 (3)	−0.0072 (2)	−0.0021 (2)	−0.0018 (2)
C11	0.0175 (3)	0.0142 (2)	0.0152 (3)	−0.0060 (2)	−0.0028 (2)	−0.0032 (2)
C12	0.0195 (3)	0.0150 (2)	0.0128 (2)	−0.0070 (2)	−0.0047 (2)	−0.0018 (2)
C13	0.0169 (3)	0.0145 (2)	0.0121 (2)	−0.0066 (2)	−0.0024 (2)	−0.0032 (2)
C14	0.0157 (3)	0.0156 (2)	0.0125 (2)	−0.0058 (2)	−0.0036 (2)	−0.0034 (2)
C15	0.0158 (3)	0.0156 (2)	0.0150 (3)	−0.0046 (2)	−0.0048 (2)	−0.0036 (2)
C16	0.0176 (3)	0.0162 (3)	0.0137 (2)	−0.0059 (2)	−0.0055 (2)	−0.0027 (2)
C17	0.0197 (3)	0.0164 (3)	0.0120 (2)	−0.0065 (2)	−0.0035 (2)	−0.0033 (2)
C18	0.0182 (3)	0.0204 (3)	0.0161 (3)	−0.0019 (2)	−0.0052 (2)	−0.0065 (2)
C19	0.0164 (3)	0.0206 (3)	0.0151 (3)	−0.0035 (2)	−0.0051 (2)	−0.0057 (2)
C20	0.0305 (4)	0.0256 (3)	0.0223 (3)	−0.0114 (3)	0.0047 (3)	−0.0130 (3)
C21	0.0256 (3)	0.0196 (3)	0.0213 (3)	−0.0121 (3)	−0.0028 (3)	−0.0029 (3)
C22	0.0220 (3)	0.0194 (3)	0.0239 (3)	−0.0048 (2)	−0.0029 (3)	−0.0100 (3)
C23	0.0250 (3)	0.0172 (3)	0.0187 (3)	−0.0082 (2)	−0.0058 (2)	0.0011 (2)

### Geometric parameters (Å, °)

**Table:**

O1—C1	1.3680 (8)	C11—C22	1.5314 (10)
O1—C13	1.3798 (8)	C11—C23	1.5324 (10)
O2—C5	1.2348 (9)	C11—C12	1.5371 (9)
O3—C9	1.2273 (9)	C12—C13	1.4897 (9)
O4—C17	1.3658 (8)	C12—H12A	0.9700
O4—H1O4	0.851 (17)	C12—H12B	0.9700
C1—C6	1.3498 (9)	C14—C15	1.3931 (9)
C1—C2	1.4930 (9)	C14—C19	1.3974 (10)
C2—C3	1.5349 (9)	C15—C16	1.3922 (9)
C2—H2A	0.9700	C15—H15A	0.9300
C2—H2B	0.9700	C16—C17	1.3948 (10)
C3—C20	1.5272 (10)	C16—H16A	0.9300
C3—C21	1.5280 (10)	C17—C18	1.3950 (10)
C3—C4	1.5330 (10)	C18—C19	1.3934 (10)
C4—C5	1.5116 (10)	C18—H18A	0.9300
C4—H4A	0.9700	C19—H19A	0.9300
C4—H4B	0.9700	C20—H20A	0.9600
C5—C6	1.4628 (9)	C20—H20B	0.9600
C6—C7	1.5152 (9)	C20—H20C	0.9600
C7—C8	1.5096 (9)	C21—H21A	0.9600
C7—C14	1.5302 (9)	C21—H21B	0.9600
C7—H7A	0.9800	C21—H21C	0.9600
C8—C13	1.3445 (9)	C22—H22A	0.9600
C8—C9	1.4773 (9)	C22—H22B	0.9600
C9—C10	1.5177 (10)	C22—H22C	0.9600
C10—C11	1.5354 (10)	C23—H23A	0.9600
C10—H10A	0.9700	C23—H23B	0.9600
C10—H10B	0.9700	C23—H23C	0.9600
			
C1—O1—C13	118.25 (5)	C10—C11—C12	108.40 (6)
C17—O4—H1O4	108.2 (11)	C13—C12—C11	112.63 (5)
C6—C1—O1	122.95 (6)	C13—C12—H12A	109.1
C6—C1—C2	125.29 (6)	C11—C12—H12A	109.1
O1—C1—C2	111.76 (5)	C13—C12—H12B	109.1
C1—C2—C3	112.06 (5)	C11—C12—H12B	109.1
C1—C2—H2A	109.2	H12A—C12—H12B	107.8
C3—C2—H2A	109.2	C8—C13—O1	123.08 (6)
C1—C2—H2B	109.2	C8—C13—C12	125.63 (6)
C3—C2—H2B	109.2	O1—C13—C12	111.29 (5)
H2A—C2—H2B	107.9	C15—C14—C19	118.00 (6)
C20—C3—C21	109.14 (6)	C15—C14—C7	121.39 (6)
C20—C3—C4	110.88 (6)	C19—C14—C7	120.59 (6)
C21—C3—C4	109.50 (6)	C16—C15—C14	121.28 (6)
C20—C3—C2	111.08 (6)	C16—C15—H15A	119.4
C21—C3—C2	109.08 (6)	C14—C15—H15A	119.4
C4—C3—C2	107.12 (6)	C15—C16—C17	120.04 (6)
C5—C4—C3	114.58 (6)	C15—C16—H16A	120.0
C5—C4—H4A	108.6	C17—C16—H16A	120.0
C3—C4—H4A	108.6	O4—C17—C16	122.64 (6)
C5—C4—H4B	108.6	O4—C17—C18	117.87 (6)
C3—C4—H4B	108.6	C16—C17—C18	119.48 (6)
H4A—C4—H4B	107.6	C19—C18—C17	119.72 (7)
O2—C5—C6	119.90 (6)	C19—C18—H18A	120.1
O2—C5—C4	120.92 (6)	C17—C18—H18A	120.1
C6—C5—C4	119.14 (6)	C18—C19—C14	121.44 (6)
C1—C6—C5	117.69 (6)	C18—C19—H19A	119.3
C1—C6—C7	122.86 (6)	C14—C19—H19A	119.3
C5—C6—C7	119.44 (6)	C3—C20—H20A	109.5
C8—C7—C6	108.86 (5)	C3—C20—H20B	109.5
C8—C7—C14	111.06 (5)	H20A—C20—H20B	109.5
C6—C7—C14	110.30 (5)	C3—C20—H20C	109.5
C8—C7—H7A	108.9	H20A—C20—H20C	109.5
C6—C7—H7A	108.9	H20B—C20—H20C	109.5
C14—C7—H7A	108.9	C3—C21—H21A	109.5
C13—C8—C9	118.04 (6)	C3—C21—H21B	109.5
C13—C8—C7	122.91 (6)	H21A—C21—H21B	109.5
C9—C8—C7	119.05 (6)	C3—C21—H21C	109.5
O3—C9—C8	120.20 (6)	H21A—C21—H21C	109.5
O3—C9—C10	120.86 (6)	H21B—C21—H21C	109.5
C8—C9—C10	118.87 (6)	C11—C22—H22A	109.5
C9—C10—C11	115.38 (6)	C11—C22—H22B	109.5
C9—C10—H10A	108.4	H22A—C22—H22B	109.5
C11—C10—H10A	108.4	C11—C22—H22C	109.5
C9—C10—H10B	108.4	H22A—C22—H22C	109.5
C11—C10—H10B	108.4	H22B—C22—H22C	109.5
H10A—C10—H10B	107.5	C11—C23—H23A	109.5
C22—C11—C23	109.53 (6)	C11—C23—H23B	109.5
C22—C11—C10	110.09 (6)	H23A—C23—H23B	109.5
C23—C11—C10	110.01 (6)	C11—C23—H23C	109.5
C22—C11—C12	110.94 (6)	H23A—C23—H23C	109.5
C23—C11—C12	107.83 (5)	H23B—C23—H23C	109.5
			
C13—O1—C1—C6	−2.38 (10)	C7—C8—C9—C10	174.00 (6)
C13—O1—C1—C2	177.69 (6)	O3—C9—C10—C11	161.59 (7)
C6—C1—C2—C3	−25.58 (10)	C8—C9—C10—C11	−21.61 (9)
O1—C1—C2—C3	154.34 (6)	C9—C10—C11—C22	−73.51 (7)
C1—C2—C3—C20	−69.86 (8)	C9—C10—C11—C23	165.69 (6)
C1—C2—C3—C21	169.79 (6)	C9—C10—C11—C12	48.00 (8)
C1—C2—C3—C4	51.36 (7)	C22—C11—C12—C13	72.05 (7)
C20—C3—C4—C5	69.49 (8)	C23—C11—C12—C13	−168.01 (6)
C21—C3—C4—C5	−170.02 (6)	C10—C11—C12—C13	−48.94 (7)
C2—C3—C4—C5	−51.86 (7)	C9—C8—C13—O1	−175.73 (6)
C3—C4—C5—O2	−157.76 (6)	C7—C8—C13—O1	4.22 (10)
C3—C4—C5—C6	24.75 (9)	C9—C8—C13—C12	4.00 (10)
O1—C1—C6—C5	175.35 (6)	C7—C8—C13—C12	−176.05 (6)
C2—C1—C6—C5	−4.74 (10)	C1—O1—C13—C8	3.14 (10)
O1—C1—C6—C7	−5.68 (10)	C1—O1—C13—C12	−176.62 (5)
C2—C1—C6—C7	174.24 (6)	C11—C12—C13—C8	25.26 (9)
O2—C5—C6—C1	−172.19 (6)	C11—C12—C13—O1	−154.98 (6)
C4—C5—C6—C1	5.34 (9)	C8—C7—C14—C15	134.11 (6)
O2—C5—C6—C7	8.80 (10)	C6—C7—C14—C15	−105.11 (7)
C4—C5—C6—C7	−173.67 (6)	C8—C7—C14—C19	−47.61 (8)
C1—C6—C7—C8	11.35 (9)	C6—C7—C14—C19	73.17 (8)
C5—C6—C7—C8	−169.70 (6)	C19—C14—C15—C16	0.67 (10)
C1—C6—C7—C14	−110.74 (7)	C7—C14—C15—C16	178.99 (6)
C5—C6—C7—C14	68.21 (8)	C14—C15—C16—C17	1.03 (10)
C6—C7—C8—C13	−10.62 (9)	C15—C16—C17—O4	177.02 (6)
C14—C7—C8—C13	111.01 (7)	C15—C16—C17—C18	−2.02 (10)
C6—C7—C8—C9	169.33 (6)	O4—C17—C18—C19	−177.79 (6)
C14—C7—C8—C9	−69.04 (8)	C16—C17—C18—C19	1.30 (11)
C13—C8—C9—O3	170.78 (7)	C17—C18—C19—C14	0.43 (11)
C7—C8—C9—O3	−9.17 (10)	C15—C14—C19—C18	−1.40 (10)
C13—C8—C9—C10	−6.04 (9)	C7—C14—C19—C18	−179.74 (6)

### Hydrogen-bond geometry (Å, °)

**Table:**

*D*—H···*A*	*D*—H	H···*A*	*D*···*A*	*D*—H···*A*
O4—H1O4···O2^i^	0.851 (18)	1.910 (17)	2.7423 (9)	165.6 (17)

Symmetry codes: (i) −*x*, −*y*+2, −*z*+1.
